# Causes and Effects Contributing to Sudden Death in Epilepsy and the Rationale for Prevention and Intervention

**DOI:** 10.3389/fneur.2020.00765

**Published:** 2020-07-31

**Authors:** Mark Stewart, Joshua B. Silverman, Krishnamurthi Sundaram, Richard Kollmar

**Affiliations:** ^1^Department of Neurology, State University of New York Health Sciences University, Brooklyn, NY, United States; ^2^Department of Physiology & Pharmacology, State University of New York Health Sciences University, Brooklyn, NY, United States; ^3^Department of Otolaryngology, North Shore Long Island Jewish Medical Center, New Hyde Park, NY, United States; ^4^Department of Otolaryngology, State University of New York Health Sciences University, Brooklyn, NY, United States; ^5^Department of Cell Biology, State University of New York Health Sciences University, Brooklyn, NY, United States

**Keywords:** apnea, laryngospasm, SUDEP, airway obstruction, respiratory arrest

## Abstract

Sudden unexpected death in epilepsy (SUDEP) claims the lives of one in every thousand epileptic patients each year. Autonomic, cardiac, and respiratory pieces to a mechanistic puzzle have not yet been completely assembled. We propose a single sequence of causes and effects that unifies disparate and competitive concepts into a single algorithm centered on ictal obstructive apnea. Based on detailed animal studies that are sometimes impossible in humans, and striking parallels with a growing body of clinical examples, this framework (1) accounts for the autonomic, cardiac, and respiratory data to date by showing the causal relationships between specific elements, and (2) highlights specific kinds of data that can be used to precisely classify various patient outcomes. The framework also justifies a “near miss” designation to be applied to any cases with evidence of obstructive apnea even, and perhaps especially, in individuals that do not require resuscitation. Lastly, the rationale for preventative oxygen therapy is demonstrated. With better mechanistic understanding of SUDEP, we suggest changes for detection and classification to increase survival rates and improve risk stratification.

## Introduction: Physiological Background and Key Concepts

Reviews of preclinical and clinical data on autonomic, cardiovascular, and respiratory contributions to sudden unexpected death in epilepsy (SUDEP) have captured the progress made toward understanding this important aspect of epilepsy (1–7). Whereas, the mechanisms for SUDEP remain unknown, the main categories of potential mechanism are (1) autonomic derangements, as these are the critical link between seizure activity and the rest of the body (2) lethal cardiac events, which can link epileptogenesis and cardiac risk among the channelopathies, and (3) apnea, which may result from seizure spread to brainstem or a catastrophic failure of brainstem circuits. The key challenge has been to demonstrate which of these is/are responsible for SUDEP given the “unexpected” nature of cases and the limitations on physiological monitoring during events.

Humans and animals have extensive pathways involving insular cortex, subiculum, and amygdala that permit seizure spread to reach hypothalamus and brainstem autonomic preganglionic and premotor neurons and thus impact all body systems with autonomic innervation (8). Changes in cardiac, respiratory, gastrointestinal, and genitourinary function before, during and after a seizure are well-known from clinical and preclinical data (2, 9–15). Significant autonomic effects of seizures occur more commonly in association with generalized tonic-clonic seizures or partial seizures originating in the temporal lobe (16–18).

In contrast to the direct ictal activation of the autonomic nervous system (ANS), there can be autonomic activity that is secondary to other ictal phenomena (e.g., hypoxemia from obstructive apnea) (13, 19, 20). Such autonomic activity is a “normal” response to protect core blood flow during a survival threat.

Repeated organ stress caused by recurring seizures or parallel pathophysiological processes as in the heritable channelopathies can lead to sustained autonomic abnormalities that impact the direct or indirect responses to seizure activity. Some have argued that an abnormal autonomic baseline is essential for the extreme physiological events leading to sudden death (21–26).

Pathways exist for seizure activity to impact respiratory rhythm generation and motor output (27–30). Reports of ictal tachypnea, bradypnea, and apnea all point to an impact of seizure activity on respiratory physiology (3, 31–35) and thereby a role in ictal oxygen desaturation (36–39).

Ictal airway obstruction has been reported in humans (40–44), and our group demonstrated laryngospasm as the basis for ictal obstructive apnea (defined as periods of no airflow with evidence of inspiratory effort) using continuous laryngoscopy, recurrent laryngeal nerve recordings, plethysmography, ECG, and EEG in a rat model (34). Obstructive apnea (OA) was accompanied by pronounced hypoxemia, followed by bradycardia, respiratory arrest, and eventually death (34, 45). Further evidence of airway obstruction as part of the SUDEP mechanism is the fact that pulmonary edema is often found at autopsy in SUDEP cases (46–50).

Ictal central apnea (defined as periods of no airflow and no evidence of respiratory effort) has been demonstrated with recordings that can distinguish central apnea from OA or respiratory arrest (34, 51–53). During ictal central apnea, the central respiratory rhythm generation continues and the respiratory motor output is inhibited in the same manner as during the apnea that occurs with voluntary breath holding or the diving response, a complex reflex that includes apnea and co-activation of the divisions of the ANS (51, 54–56). A remarkable example is the “central” apnea associated with amygdala stimulation (57, 58). The absence of “stress” during amygdala-evoked apnea and the minimal oxygen desaturation is consistent with spontaneous ictal central apnea events having resemblance to the diving response. Mouse deaths from audiogenic seizures have been suspected to involve central apnea or respiratory arrest due to brainstem disruption, particularly brainstem circuits involving serotonergic neurons (27, 29, 32, 59–64), but we showed deaths to include obstructive apnea leading to respiratory arrest (65).

Lethal arrhythmias appear to be less common. Cases of ventricular fibrillation (VF) arising from seizure activity (66, 67) or seizure-induced takotsubo cardiomyopathy (68) have been reported. Whereas, the most common cause of VF in humans is regional cardiac ischemia in the setting of myocardial infarction, global hypoxemia, such as may occur during asystole or apnea, has also been implicated in severe tachyarrhythmias (69, 70). We have shown in rats that entry into ventricular tachycardia and ventricular fibrillation could occur spontaneously under narrow conditions of moderate, but not severe hypoxia, sympathetic overdrive, and minimal vagal activity (71, 72). Whereas, VF is certainly one path to SUDEP, the existing literature indicates that it is uncommon.

## Proposed Sudep Mechanism Accounts For Causes and Effects

**Figure 1:**
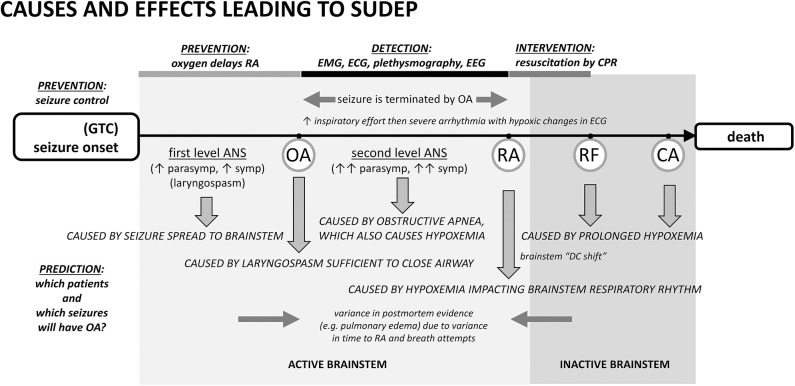
Proposed cascade of autonomic, cardiac, and respiratory causes and effects leading to sudden death following a seizure with points of prevention, detection, and intervention. Seizure spread to brainstem laryngomotor neurons causes laryngospasm, which can be sufficient in a minority of cases for obstructive apnea (OA). OA drives additional autonomic co-activation and is associated with a rapid oxygen desaturation. Desaturation leads to respiratory arrest (RA), respiratory failure (RF), and ultimately cardiac arrest (CA). The best-established form of prevention is seizure control. An alternative prevention is to provide oxygen at the beginning of the seizure or earlier. Oxygen can delay the time to RA long enough to permit the seizure to end spontaneously even if the airway is transiently occluded by ictal laryngospasm. In the detection period, EMG or inductance plethysmography can provide evidence of inspiratory exertion during obstructive apnea. EEG can show that the seizure was aborted. Pre- and post-mortem evidence of airway occlusion is variable because consequences such as pulmonary edema will depend upon the amount of time the airway was obstructed and the frequency and amplitude of inspiratory attempts. ANS, autonomic nervous system.

Two recent lines of research enable us to propose a comprehensive mechanistic sequence for the majority of SUDEP cases (Figure 1). The first was the report of results from the MORTEMUS study (1), which summarized the range of autonomic, cardiac, and respiratory data between seizure onset and death from the rare human SUDEP cases that could be clearly identified as such and at the same time were accompanied by recordings of vital signs. This critical consensus established the sequence of clinical “landmarks” in SUDEP cases. The second was extensive work with invasive and non-invasive monitoring in rodents that showed how OA occurs during seizures, how OA serves as the link between a seizure and respiratory arrest (RA), and how non-invasive measures can be used to interpret human data. Demonstrations that ictal OA can be due to laryngospasm (34), that inspiratory effort can be detected with EMG (45) or inductance plethysmography (73), and even that a surrogate airway protects against death in a widely-studied mouse model of SUDEP (65) collectively argue that OA is part of a common mechanism for SUDEP. These data permit events associated with a seizure to be defined as causes or effects.

In our opinion, the sequence begins with a generalized seizure. Seizure generalization to brainstem autonomic and respiratory areas is the cause of “first level” autonomic co-activation, irregular ventilation, and laryngospasm producing partial airway occlusion. Autonomic co-activation is a source of physiological variance. Heart rate, for example, will be altered and the observed increase or decrease in ictal rate depends upon the relative levels of the autonomic components [as well as the baseline heart rate (74)]. Laryngomotor neurons are driven by seizure spread (34) and the resulting “convulsive” movement of the vocal folds (laryngospasm) occurs throughout the seizure, but an adequate airway is usually maintained. As the seizure ends spontaneously, the drive to alter autonomic and respiratory activity is eliminated (12, 74).

Occasionally laryngospasm is sufficient to cause OA (34, 52). OA is associated with intense effort to inspire, rapid desaturation, and a significant “second level” autonomic co-activation to protect core blood flow. EMG evidence of the unproductive inspiratory effort can appear in ECG and EEG records (1, 45). This measure has been used in studies of obstructive sleep apnea (75–77) and can been seen in recordings from elite apneists (e.g., breath-holding divers) (78), where stertorous breathing is not a confound. Inductance plethysmography in epilepsy patients also shows the inspiratory effort (73).

**Figure 2:**
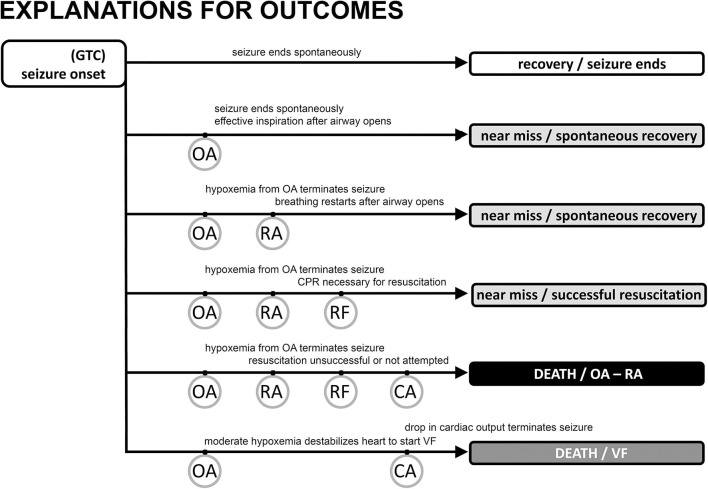
Possible outcomes following generalized tonic clonic seizure activity with major “landmarks.” The vast majority of seizures are associated with a spontaneous end of the seizure, no evidence of obstructive apnea, and a rapid return of autonomic, cardiac, and respiratory function to baseline levels (track 1). Obstructive apnea (OA) can occur, resulting in three types of near miss outcome (tracks 2, 3, 4) or sudden death (track 5). If the seizure ends spontaneously during OA (track 2) there may be evidence of inspiratory effort, but the EEG will show a normal pattern of decreasing frequency/increasing amplitude associated with the seizure ending on its own. Preventative oxygen treatment will successfully move any case to track 2. Seizure activity will be aborted in tracks 3 and 4 evidenced by EEG frequency increase/amplitude decrease due to a lack of brain blood flow and oxygen. The difference between tracks 3 and 4 is whether resuscitation is necessary (track 4) because of the inability to spontaneously recover breathing, i.e., respiratory failure (RF). SUDEP results from the events in tracks 5 and 6 where cardiac arrest (CA) is the endpoint. Track 5 is the sequence described in the text and shown in greater detail in Figure 1. CA is hypoxic cardiac failure in track 5. Track 6 is the outcome of seizure-induced ventricular fibrillation and a global hypoxemia trigger due to OA or asystole with or without OA.

Seizures can end in two different ways after a period of OA has started. In the first, most common way, seizures end spontaneously, i.e., on their own. The stimulus for laryngospasm ends as the seizure ends. Alternatively, the hypoxemia and decreased cardiac output associated with the autonomic changes can abort the ongoing seizure activity (34, 45, 79, 80) by starving it of blood flow and oxygen. Seizure termination by asystole has been specifically noted in the clinical literature (81–83). Once aborted, recovery of baseline autonomic, cardiac, and respiratory function occurs because the seizure stimulus was removed. The full set of outcomes is illustrated in Figure 2. Based on our experience with verified OA (34), controlled airway occlusion (45), or asystole (80), the EEG can differentiate between seizures that end spontaneously and those that are aborted [see (84) for a mechanistic example in a different context]. Aborted seizure activity ends with a decrease in EEG amplitude and modest increase in EEG frequency, not the typical increase in amplitude and associated decrease in frequency.

In the second, rarer, but more dangerous way, seizure activity can persist and thus OA can last to the point of respiratory arrest (RA), defined as the point at which attempts to inspire cease (45, 53). This is a critical concept because it is the point at which spontaneous recovery is in jeopardy, and intervention by personnel other than the person experiencing the seizure may be necessary. Based on our work, the point of RA corresponds to the onset of “terminal apnea” as described in the MORTEMUS data (1). RA is distinct from respiratory failure (RF), which is defined as the point at which attempts to inspire are no longer possible. If the airway opens after the point of RA, but before RF, spontaneous recovery of respiration can sometimes occur (53). Apneic oxygenation is possible once the airway re-opens due to glottic relaxation (85), and this may account for the spontaneous recovery of respiration. Postural or positional factors can contribute to a compromised airway and block spontaneous recovery (86, 87). Based on this conceptual framework, we suggest that any case involving OA should be identified as a near-miss case, irrespective of whether the seizure ended spontaneously or was aborted, or whether the individual required resuscitation for recovery (Figure 2).

Cardiopulmonary resuscitation is known to be effective within a short time after the onset of terminal apnea/respiratory arrest (1). In our experience, a majority of cases that reach the point of RA, without resuscitation attempts, progress to respiratory failure and death (34). Cases progressing to respiratory failure will show hypoxic cardiac failure as the final sign of life.

Postictal EEG suppression (PGES) or brain shutdown, suggested as a cause of brainstem dysfunction and death (26, 88, 89) is not a cessation of brain activity (90, 91). Rather, it reflects the termination of seizure activity by hypoxemia and decreased cardiac output (12, 34, 80). The work on brainstem depolarization (92) demonstrates that this form of spreading depression likely accounts for the development of respiratory failure after reaching the point of respiratory arrest. The time to develop, the time of occurrence, and the time that would be necessary for resolution all point to brainstem depolarization occurring as a result of hypoxemia and after the point of respiratory arrest (52, 93).

Lastly, any seizure-driven cascade of events is further complicated by the possibility that (and predictive opportunity that arises when) seizures impact an abnormal background physiology due to repeated seizures, genetic variation (30, 94–97), pharmacotherapy, or other causes. None of our proposed sequence of events from seizure onset to death depends upon an abnormal background physiology.

## Discussion: Application In Prevention, Intervention, and Classification

Two forms of prevention have been discussed in the literature. The most straightforward prevention is seizure control, which avoids the sequence of life-threatening events (98). A second strategy is to expose the individual to oxygen as early as possible. Oxygen, even for a short time prior to the onset of OA, delays the time to RA (53, 99, 100) and thus permits spontaneous seizure termination even if OA is present. Based on our data and proposed sequence of events, a critical preventative intervention is to enrich the inspired oxygen as near the onset of a seizure as possible, without waiting for evidence of obstructive apnea (oxygen will NOT prevent obstructive apnea, but will help the individual to survive an episode of OA). This should delay RA and prevent SUDEP (Figure 2). Oxygen would also minimize the potential for VF. We propose that any case with evidence of OA should be counted as a near miss so that in these higher risk patients, oxygen will be applied earlier, potentially preventing SUDEP.

CPR has been shown to be an effective for resuscitation within an adequate time window (1). During OA, artificial ventilation will be possible only after the laryngospasm relaxes to permit airflow. Chest compressions are important because cardiac contractility is minimal after the point of respiratory arrest (34). There is a vital race to start CPR before irreversible respiratory failure.

Risk stratification and prediction of life-threatening events remain a challenge. Post convulsive central apnea (PCCA) (70, 101) has been suggested as a predictive biomarker to stratify risk of SUDEP among epilepsy patients (102). PCCA in our framework would describe the period of time from respiratory arrest to spontaneous or assisted recovery of respiration. Prior to RA, OA accounts for the absence of airflow. PCCA is clear evidence of near miss status and this explains its predictive value.

Still missing is an answer to the question of why only some seizures have laryngospasm sufficient to cause OA. Fortunately, indicators of OA exist (e.g., our biomarker or inductance plethysmography) and can be used for risk stratification even if oxygen is delivered at the start of every seizure to prevent RA. Further complicating the classification of cases and prediction, but not complicating our mechanistic sequence, is whether the individual experiencing the seizure that causes sudden death is considered epileptic (103), i.e., what if the first seizure you have is the one that causes sudden death?

In summary, (1) we propose that obstructive apnea is the critical mechanistic link between seizure activity and respiratory and cardiac failure in the majority of SUDEP cases, (2) we recommend modifying the “near SUDEP” definition (104) to include any individuals with a near miss event because these indicate that the patient is prone to seizure-induced obstructive apnea and thus at increased risk for SUDEP, and (3) we argue that early oxygen exposure is a rational preventative step that can significantly reduce SUDEP rates. Based on our improved mechanistic understanding, we suggest changes for detection and classification of SUDEP patients to increase their survival and enhance their risk stratification.

## Author Contributions

All authors listed have made a substantial, direct and intellectual contribution to the work, and approved it for publication.

## Conflict of Interest

The authors declare that the research was conducted in the absence of any commercial or financial relationships that could be construed as a potential conflict of interest.
